# Brainstem Anaesthesia after Retrobulbar Block

**DOI:** 10.1515/med-2019-0025

**Published:** 2019-03-02

**Authors:** Ivan Kostadinov, Andrej Hostnik, Barbara Cvenkel, Iztok Potočnik

**Affiliations:** 1 Clinical Department of Anaesthesiology and Intensive Therapy, University Medical Centre Ljubljana, Zaloška 7, 1000 Ljubljana, Slovenia; 2 Clinical Department of Anaesthesiology and Intensive Therapy, University Medical Centre Ljubljana, Ljubljana, Slovenia; 3 Department of Ophthalmology, University Medical Centre Ljubljana, Ljubljana, Slovenia; 4 Medical Faculty, University of Ljubljana, Ljubljana, Slovenia; 5 Department of Anaesthesiology and Intensive Care, Institute of Oncology Ljubljana, Ljubljana, Slovenia

**Keywords:** Retrobulbar block complications, Brainstem anaesthesia, Local anaesthetic toxicity

## Abstract

Regional anaesthesia techniques in ophthalmology are usually utilized for day case surgery. During various procedures, profound akinesia of the eye and anaesthesia of the surgical site are required, both of which are achieved with retrobulbar block. Due to the anatomy of the eye, life-threatening complications are possible. An 82-year-old female with secondary post-herpetic uveitic glaucoma of the right eye presented at the Department of Ophthalmology for an elective trans-scleral laser cyclophotocoagulation. She was given a retrobulbar block to the right eye with 2 mL of 0.5% levobupivacaine and 2 mL of 2% lidocaine. The procedure was technically performed without any issues. 2-3 minutes after the injection she became lethargic and 5 minutes later she lost consciousness and developed severe hypotension with bradycardia and respiratory arrest. She was successfully intubated and resuscitated, using mechanical ventilation, vasoactive medications, fluid therapy and intravenous lipid emulsion. There are three mechanisms for local anaesthetic (LA) to reach the central nervous system after a retrobulbar block: systemic absorption of LA, direct intra-arterial injection and retrograde flow into the cerebral circulation, and injecting LA into the subdural space via puncturing the dural optic nerve sheath, the latter being most common. The clinical picture of our patient was very consistent with subdural anaesthesia after exposure of the pons, midbrain and cranial nerves to LA, i.e. brainstem anaesthesia. Following appropriate life support measures taken in our case, there was a successful outcome. To minimize the chance for brainstem anaesthesia after retrobulbar block, we recommend low volume with low concentration of LA and block performance by an experienced ophthalmologist or anaesthesiologist with proper technique. Patients receiving retrobulbar anaesthesia should be carefully monitored at least 20 minutes after the block. Life support equipment should be available before performing retrobulbar block.

## 1 Introduction

The advantages of regional anaesthesia over general anaesthesia in terms of safety, efficacy, and patient satisfaction are well known. In ophthalmic surgery, regional anaesthesia techniques usually utilized for day case surgery include peribulbar, retrobulbar and sub-Tenon’s block [1, 2]. During the procedure, profound akinesia of the eye and anaesthesia of the surgical site are required, both of which are achieved with a retrobulbar block. Injection of 2 to 4 mL of anaesthetic solution is usually made between the extraocular muscles [3]. Due to the anatomy of the eye, life-threatening complications such as retrobulbar haemorrhage, perforation of the globe, globe ischemia, central retinal artery occlusion, optic nerve damage, strabismus, myotoxicity, intra-arterial injection, brainstem anaesthesia (BSA) and cardiopulmonary arrest are possible [4, 5, 6].

## 2 Case report

An 82-year-old female with secondary post-herpetic uveitic glaucoma of the right eye presented at the Department of Ophthalmology for an elective trans-scleral laser cyclophotocoagulation. She had a history of chronic heart failure, arterial hypertension, hyperlipidemia, depression and Hodgkin’s lymphoma in remission. Her regular medications included acetylsalicylic acid, bisoprolol, isosorbide mononitrate, telmisartan, atorvastatin and escitaloprame. The patient denied having any allergies. On preoperative assessment she weighed 62 kg, her height was 162 cm and her blood pressure (BP) was 166/83 mmHg with 70/min heart rate (HR). Physical examination was unremarkable.

The patient’s orbit was anatomically normal. She was given a retrobulbar block to the right eye with 2 mL of 0.5% levobupivacaine and 2 mL of 2% lidocaine. No mydriatic agent was used. She did not move her eye during the procedure. Aspiration for blood was negative. Upon injection, no resistance was felt. 2-3 minutes after the injection, she started yawning and feeling progressively drowsy; anaesthesiology team was immediately called. 5-8 minutes after the injection she became unresponsive to verbal and tactile stimuli; her Glasgow Coma Scale (GCS) score was 3. On arrival of the team she had developed bradycardia with hypotension, her HR was 40/min, BP 50/30 mmHg and blood oxygen saturation (SpO2) 85%. 10 mg of ephedrine was promptly administered intravenously (i.v.) with no significant effect, continued by 3 i.v. doses of 0.1 mg adrenaline 2 minutes later. Concurrently the patient developed respiratory arrest, her SpO2 had fallen to 54%, BP was 163/100 mmHg with HR 93/min. A bolus of 200 mL intravenous lipid emulsion (ILE) was given. Anaesthesia was induced with 50 mg of propofol, the patient was intubated and mechanically ventilated, and her vitals had stabilized (SpO2 98%, BP 113/60 mmHg, HR 92/min). She was additionally sedated with 5 mg of midazolam. Because of persistent hypotension (RR 92/58), 500 mL of hydroxyethyl starch (HES 130/0.4) in isotonic sodium chloride solution was also administered.

Surgery was cancelled and the patient was relocated to the ICU. She was additionally sedated with midazolam and propofol, then gradually shifted through supportive ventilation techniques to spontaneous ventilation and extubated 6 hours after admission. There was no need for vasoactive support, oxygen or fluid replacement therapy. Blood test results were within normal range. Her BP was 140/60 mmHg, HR 71/min, SpO2 95%, without additional oxygen. Her GCS score was 15. The next day she was discharged to the Department of Ophthalmology, where the intended procedure was performed under general anaesthesia. The patient was discharged home 6 days later, she was stable and had not suffered any consequences from the adverse reaction.

Informed consent has been obtained from the patient to use the data presented in this case report.

The research has been complied with all the relevant national regulations, institutional policies and in accordance the tenets of the Helsinki Declaration.

## 3 Discussion

Retrobulbar injection of an anaesthetic is a generally safe and effective method of obtaining anaesthesia and akinesia for ocular surgery [7]. Brainstem anaesthesia is a well-known but fortunately rare complication of the retrobulbar block. This complication can have devastating consequences such as apnea and cardiac arrest. In a retrospective study of 6000 retrobulbar blocks, Nicoll et al. reported 0.27% incidence of central nervous system involvement [8]. Other studies report similar low incidence of 0.09% and 0.79% [9, 10].

There are three mechanisms for the local anaesthetic to reach the central nervous system. The first mechanism is via systemic absorption of LA. The second is after direct intra-arterial injection of local anaesthetic and retrograde flow into the cerebral circulation. The third mechanism is by puncturing the dural optic nerve sheath and injecting LA into the subdural space causing its accumulation around the brainstem [11]. The third mechanism has been cited as the most likely scenario in cases where central nervous system adverse symptomatology occurs after the retrobulbar LA application.

In the literature we can find some rare cases of allergic reaction to LA. LA common adverse reactions and clinical symptoms often correspond to anaphylaxis with tachycardia, hypotension and subjective feelings of weakness, heat or vertigo. The pathomechanism of immediate hypersensitivity reaction to LA is largely unknown – it is commonly regarded as ‘pseudo-allergic’ or ‘non-immune type’ of anaphylaxis [12]. Since no symptoms such as upper airway oedema and bronchospasm, skin rush and pruritus were present in our case, we can easily rule out anaphylaxis as a possible complication.

Acute coronary syndrome in the perioperative period is not rare, especially in the comorbid older population. However, in the case reported here, even though cardiovascular collapse was present at first, there were no rhythm abnormalities. Blood pressure was rapidly stabilized, and no ECG changes were present in the post-event period. Plasma troponin levels were well below the critical value. Therefore, a primary cardiovascular event such as myocardial infarction was also excluded as a reason for apnoea and loss of consciousness.

Local anaesthetic systemic toxicity (LAST) was recognized and described shortly after Koller, an Austrian ophthalmologist, first applied cocaine to the eye to perform ocular surgery in 1884. Initial contributions to the understanding of LAST was made by Tanaka et al. [13] who reported the selective blocking of cortical inhibitory synapses by lidocaine. Later on, Englesson [14] described the seizure activity in the amygdala on electroencephalography after the intravenous infusion of several different local anaesthetics in the cat model. As LAST symptoms are closely related to plasma and central nervous system (CNS) levels of the LA, the initial generalized excitatory phase of LAST, related to the increased levels of LA in the CNS, results from blocking the inhibitory pathways in the amygdala and hippocampus. This allows excitatory neurons to function unopposed [15, 16]. For LAST to appear, toxic plasma and CNS levels of LA need to be reached because of either an unintentional intravascular injection of the LA or administration of large amount of LA. Brown et al. [17] suggested that unintentional intravascular injection of the LA, rather than uptake of excessive dose administered during the block, was the main reason for LAST. This is supported by the buffering capacity of the lungs up-taking up to 90% of the LA administered [18]. Before generalized tonic-clonic convulsions occur, as a CNS manifestation of LAST, patients may experience various symptoms and signs such as: light-headedness, dizziness, numbness of the tongue, metal taste in the mouth, tinnitus, muscle twitching, shivering, and tremor. CNS toxic responses occur at lower blood levels than the cardiovascular system (CVS) toxic responses. This explains why cardiovascular collapse occurs rarely compared to CNS symptomatology during LAST. Because the CVS is generally more resistant to the effects of LA than the CNS, usually 2-5 times higher plasma levels of LA are needed for CVS collapse than for the appearance of CNS toxicity [19]. In the period after LA application and before loss of consciousness, our patient did not complain of any unusual sensations, muscle cramps, tremor or anything else that could raise suspicion of inadvertent intravenous LA application. We did not observe any seizure activity. The ophthalmologist administering the retrobulbar block checked that during application the needle was not in a vessel. The amount of the LA our patient received was far below the doses required to cause LAST (lidocaine 6.4 mg/kg and levobupivacaine 1.6 mg/kg). However, inadvertent intra-arterial injection of the LA could also produce retrograde flow from the ophthalmic artery to the carotid artery. This has the potential to produce CNS symptoms. These symptoms appear immediately after intra-arterial application of the LA which was not seen in our case [21, 21].

Brainstem anaesthesia, as a form of CNS toxicity, is the most likely situation that may warrant cardiopulmonary resuscitation in ophthalmic regional anaesthesia. It is reported to occur in 1 of 350-500 intraconal LA injections [22]. Symptomatology can differ between each case of brainstem anaesthesia and it can include different combinations of confusion, unconsciousness, irregular breathing, apnoea, numb throat, dysphagia, hypotension, hypertension, bradycardia, tachycardia, cardiovascular instability, convulsions, shivering, dysarthria, and hemi-, para-, or quadriplegia [6]. In most of the cases symptoms appear in 2-10 minutes after injection. Recovery from brainstem anaesthesia usually takes 10 to 60 minutes if adequate life support is given [11].

The optic nerve and its chiasm are in close relation to the brain. The brain dural and arachnoid layers extend along the optic chiasm and the optic nerve into the orbital cavity to the posterior aspect of the sclera. As retrobulbar block is a block where the tip of the needle is placed in close vicinity to the optic nerve, it is reasonable to think that optic nerve dural puncture is possible. Evidence to support the theory of dural puncture and brainstem exposure to LA in our patient comes from radiologic and cadaveric studies. In an experimental study, Drysdale [23] injected a radiopaque dye into the intraorbital subdural space and using radiography showed diffusion of the dye posteriorly along the subdural space to the midbrain surrounding the respiratory center. In another cadaver study by Boardman et al. [24], methylene blue dye was injected into the optic nerve sheath. The blue dye was found to track along the subarachnoid space of the optic nerve sheath to the chiasmatic cistern in the middle cranial fossa. They also found that pressure generated by the injection into the optic nerve sheath (approximately 138 mmHg) was three to fourfold higher than the pressure produced by injection into the retrobulbar adipose tissue (approximately 35 mmHg). The authors concluded that any resistance encountered during retrobulbar block should serve as a warning signal, mandating needle redirection. Connection between the optic nerve and brainstem subarachnoid space was proven by another study in which the authors injected contrast agent into the subarachnoid space and demonstrated its appearance in the optic nerve sheath [25]. In a case of respiratory arrest after retrobulbar block, Kobet [26] recovered lidocaine and bupivacaine from cerebral spinal fluid obtained by lumbar puncture. Some other entities like vasovagal reaction or oculocardiac reflex can mimic the clinical scenario of brainstem anaesthesia. These conditions also appear shortly after manipulation of the eyeball. Diagnosis of central spread of LA can be positively confirmed by finding paresis of the extraocular muscles of the contralateral eye (the absence of this sign does not rule out this possibility) [8].

To minimize the possibility for optic nerve dural puncture during the retrobulbar block some authors advise the use of a blunt needle [27]. A blunt needle is less likely to penetrate the optic nerve sheath, but its insertion is more painful and traumatic. There are also reports of central spread occurring with this type of needle [21]. Maximum needle length recommended for retrobulbar block is 31 mm because serious injury is greater with the use of larger, blunt needles than fine, disposable ones [28, 29]. Bulbar position during the block performance is of paramount importance to decrease the risk for brainstem anaesthesia. Central position of the gaze is the safest. Patients are usually asked not to look upward and inward because in that position the optic nerve is most exposed to the needle tip.

Our patient had apnea, cardiovascular collapse, loss of consciousness, bilateral gaze paresis and bilateral pupillary dilation two minutes after the retrobulbar block. No convulsions were seen. This clinical picture is very consistent with the clinical picture seen in subdural anaesthesia after exposure of the pons, midbrain and cranial nerves to LA. The literature also reports cases where hypertension and tachycardia are seen instead of hypotension and bradycardia. This was explained by Hamilton [30] as a combination of vagal and carotid sinus reflex blockade. Following appropriate life support measures taken in our case, there was a successful outcome without any adverse consequences. To minimize the risk for brainstem anaesthesia after retrobulbar block we recommend low volume, low concentration of LA and block performance by an experienced ophthalmologist or anaesthesiologist with proper technique. Patients receiving retrobulbar anaesthesia should be carefully monitored for at least 20 minutes after the block. Life support equipment should be available before the block performed.

## 4 Conclusions

We have presented a patient who experienced brainstem anaesthesia with respiratory arrest and cardiocirculatory collapse as a complication of retrobulbar block. We addressed systemic complications of retrobulbar block in ophthalmic surgery, which are rare and potentially life-threatening and described the course of treatment in out particular case. In the effort to reduce incidence of such complications, we emphasize the importance of proper technique, equipment, monitoring of patient’s vital functions (blood pressure, EKG and pulse oximetry) and early recognition of adverse effects of LA.
